# SIVconsv DNA prime - TLR7/IFNα adjuvanted long peptide boost induces potent CD4+ Ab responses and protects against high dose intrarectal SIV challenge

**DOI:** 10.1186/1742-4690-9-S2-P29

**Published:** 2012-09-13

**Authors:** G Koopman, N Beenhakker, I Nieuwenhuis, G Doxiadis, P Mooij, JW Drijfhout, J Koestler, T Hanke, RE Bontrop, R Wagner, WM Bogers, CJ Melief

**Affiliations:** 1 Leiden University Medical Center, Leiden, the Netherlands; 2 University of Regensburg, Regensburg, Germany; 3 The Jenner Institute, University of Oxford, Oxford, UK; 4 BPRC, Rijswijk, the Netherlands; 5 Leiden University Medical Center Leiden, Leiden, the Netherlands

## Background

Because of the extreme variability of the HIV-1 genome, a successful vaccine has to effectively recognize diverse infecting HIV-1 strains in the population and must deal with ongoing virus escape in infected individuals. We have selected conserved regions within SIV, corresponding to HIVconsv (Letourneau PLoS ONE 2007) that show little variation across the different isolates and incorporated these in a peptide based vaccine strategy.

## Methods

In this protocol rhesus macaques were immunized subcutaneously with 46 synthetic peptides of about 30 amino acids in length, which contain epitopes that can trigger helper as well as cytotoxic T-cell responses. Peptides were formulated in Montanide ISA-720. Pegylated Type I interferon plus Imiquimod, which triggers innate responses via toll like receptor 7, were given locally at the vaccine sites as additional immune stimulatory signals. Peptides were either given alone or after two times priming with DNA expressing the same conserved regions from a RNA and codon optimized gene.

## Results

After two immunizations with only peptides strong immune responses of 2000-3000 spot forming units (SFU) per 10*6 PBMC were observed. DNA priming followed by peptide boosting generated up to 8000 SFU/10*6 PBMC, comparable to what is achieved in reported adenoviral vector systems. Predominantly CD4 T-cell responses were generated (up to 10% of cells reactive to SIVmac251), which were polyfunctional (30% IFNgamma/IL-2/TNFalpha triple production), and mainly mediated by central memory T-cells. Furthermore, peptide specific antibody responses were induced, capable of recognition of Env protein. Eight weeks after the last immunization, animals received a high dose intrarectal SIVmac251 challenge. 2/6 animals in the DNA prime/peptide boost group were protected against infection, while all 6 animals in the control group and the peptide only group were infected.

## Conclusion

Vaccine induced strong CD4/antibody focused immune responses directed against conserved regions of SIV afford protection against high dose intrarectal SIVmac251 challenge.

